# Amoxicillin 3 vs 5 days for chest-indrawing pneumonia in Malawian children

**DOI:** 10.1056/NEJMoa1912400

**Published:** 2020-07-02

**Authors:** Amy Sarah Ginsburg, Tisungane Mvalo, Evangelyn Nkwopara, Eric D. McCollum, Melda Phiri, Robert Schmicker, Jun Hwang, Chifundo B. Ndamala, Ajib Phiri, Norman Lufesi, Rasa Izadnegahdar, Susanne May

**Affiliations:** 1 Save the Children, 501 Kings Highway E #400, Fairfield, CT 06825, USA; 2 University of North Carolina Project, Lilongwe Medical Relief Fund Trust, Tidziwe Centre, Private Bag A-104, Lilongwe, Malawi; 3 Eudowood Division of Pediatric Respiratory Sciences, Department of Pediatrics, Johns Hopkins School of Medicine and Department of International Health, Johns Hopkins Bloomberg School of Public Health, 200 N Wolfe Street, Baltimore, MD, 21287, USA; 4 Department of Biostatistics, University of Washington Clinical Trial Center, Building 29, Suite 250, 6200 NE 74^th^ Street, Seattle, WA, 98115, USA; 5 Department of Pediatrics and Child Health, College of Medicine, University of Malawi, Private Bag 360, Chichiri, Blantyre, Malawi; 6 Acute Respiratory Infection and Emergency Triage Assessment and Treatment, Malawi Ministry of Health, Private Bag 65, Lilongwe, Malawi; 7 Bill & Melinda Gates Foundation, 500 Fifth Avenue N, Seattle, WA, 98109, USA

## Abstract

**BACKGROUND:**

Evidence supporting duration of antibiotic treatment for children in low-resource African settings with chest-indrawing pneumonia is lacking.

**METHODS:**

We conducted a double-blind, randomized controlled 2-arm, non-inferiority trial in Lilongwe, Malawi with follow-up for 14 days to determine whether treatment with 3 days of amoxicillin for chest-indrawing pneumonia is less effective than 5 days. HIV-uninfected children aged 2 to 59 months with chest-indrawing pneumonia were randomized to 3-or 5-day amoxicillin twice-daily. Primary endpoint was the proportion of children with treatment failure (TF) by Day 6 with a relative non-inferiority margin of 1.5 times the TF rate in the 5-day amoxicillin group. Planned secondary analyses included TF or relapse by Day 14.

**RESULTS:**

Between March 29, 2016 and April 1, 2019, 3000 children were randomly assigned to 3-day (n=1497) or 5-day (n=1503) amoxicillin. Children receiving 3-day had a 5.9% (85/1442 with outcome data) TF rate by Day 6, within the non-inferiority margin of those receiving 5-day (5.2% (75/1456) TF rate), with an adjusted absolute difference of 0.75% and 95% confidence interval (CI) -0.92%,2.41%. Among children with known Day 14 outcome, 176/1411 (12.5%) receiving 3-day and 154/1429 (10.8%) receiving 5-day had TF by Day 6 or relapse by Day 14 (absolute difference 1.7%, 95%CI -0.7%,4.1%). There were no unexpected serious adverse events.

**CONCLUSIONS:**

In HIV-uninfected African children, 3 days of amoxicillin treatment for chestindrawing pneumonia was non-inferior to 5 days. We recommend revisiting antibiotictreatment guidelines applicable to similar pediatric populations.

ClinicalTrials.gov registration: NCT02760420.

## INTRODUCTION

Approximately 920,000 children die before age 5 from pneumonia annually.^1^ There is a critical need to provide greater access to appropriate and effective treatment. Treatment of bacterial pneumonia requires an effective antibiotic used in adequate doses for an appropriate duration. Determining optimal duration of antibiotic therapy is key to ensuring effective treatment while maximizing adherence and minimizing adverse drug effects, costs, and antimicrobial resistance.

A 5-day course of oral amoxicillin at least 40mg/kg/dose twice-daily (80mg/kg/day) is recommended by World Health Organization (WHO) as first-line treatment for chestindrawing pneumonia among immune-competent children <5 years old.^2,3^ However, it is unclear whether a 5-day course of amoxicillin is necessary or if a shorter duration of treatment would be as effective. Based on studies of 3-versus 5-day oral antibiotics for fast-breathing pneumonia, WHO recommends a 3-day course of oral amoxicillin for treatment of fast-breathing pneumonia among immune-competent children <5 years old.^2,4-7^ A Cochrane review found no qualifying randomized controlled trials comparing 2-3versus 5-day intravenous antibiotics for chest-indrawing or more severe pneumonia.^8^ Few data exist to inform optimal duration of treatment for pneumonia, and no study has looked at 3-versus 5-day oral antibiotics for chest-indrawing pneumonia.^9,10^ International and national pneumonia treatment guidelines rely on expert opinion and limited and weak evidence.^10,11^ In light of the global threat of increasing antimicrobial resistance, evidence-based recommendations are needed for the optimal duration of antibiotic treatment for pneumonia. Given the paucity of African data, African-specific research in malaria-endemic settings is critical to establish optimal management of children with chest-indrawing pneumonia.

## METHODS

### Study design

The primary objective of this prospective, double-blind, randomized controlled 2-arm, non-inferiority trial was to determine whether treatment with 3-day amoxicillin in HIV-uninfected children 2-59 months of age with chest-indrawing pneumonia in a malaria-endemic region of Malawi is (null hypothesis) substantively less effective than 5-day amoxicillin. An innovative non-inferiority design was formulatedbased on the beliefthat 3-dayamoxicillincould not beexpected tobe morebeneficialthan 5-dayamoxicillin with respect totheprimary outcome oftreatment failure (TF)byDay6, but might be (alternative hypothesis) only slightly worse than 5-day amoxicillin.^12^ Children aged 2-59 months meeting the chest-indrawing pneumonia case definition (Table 1) in the outpatient departments of Kamuzu Central Hospital (KCH) and Bwaila District Hospital (BDH) in Lilongwe, Malawi were screened by study staff to determine eligibility, including testing for malaria, HIV and anemia (Table 1).

**Table 1:** 

Study definitions	
Chest-indrawing pneumonia	Cough less than 14 days or difficulty breathing AND visible indrawing of the chest wall with or without fast breathing for age
Non-severe fast-breathing pneumonia	Cough less than 14 days or difficulty breathing AND fast breathing for age
Fast breathing for age	Respiratory rate >50 breaths per minute (for children 2 to <12 months of age) or >40 breaths per minute (for children >12 months of age)
Very fast breathing for age	>70 breaths per minute (for children 2 to <12 months of age) or >60 breaths per minute (for children >12 months of age).
Severe respiratory distress	Grunting, nasal flaring, head nodding, and/or chest indrawing
Hypoxemia	Arterial oxyhemoglobin saturation (SpO2) < 90% in room air, as assessed non-invasively by a pulse oximeter
World Health Organization (WHO) Integrated Management of Childhood Illness (IMCI) general danger signs	Lethargy or unconsciousness, convulsions, vomiting everything, inability to drink or breastfeed
Severe acute malnutrition	Weight for height/length < -3 SD, mid-upper arm circumference (MUAC) <11·5 cm, or peripheral edema
Severe malaria	Positive malaria rapid diagnostic test (mRDT) with any WHO IMCI general danger sign, stiff neck, abnormal bleeding, clinical jaundice, or hemoglobinuria
HIV-1 exposure	Children <24 months of age with a HIV-infected mother
Serious adverse event	Adverse event that: Results in death Is life threatening Requires inpatient hospitalization or prolongation of existing hospitalization Results in persistent or significant disability/incapacity Is a medical event, based on appropriate medical judgment, that may jeopardize the health of the participating child or require medical or surgical intervention to prevent 1 of the outcomes listed
Eligibility criteria	
Inclusion criteria	2-59 months of age Cough <14 days or difficulty breathing Visible indrawing of the chest wall with or without fast breathing for age Ability and willingness of child’s caregiver to provide informed consent and to be available for follow-up for the planned duration of the study, including accepting a home visit if he/she fails to return for a scheduled study follow-up visit
Exclusion criteria	Severe respiratory distress Hypoxemia Resolution of chest indrawing after bronchodilator challenge, if wheezing at screening examination WHO IMCI general danger signs Stridor when calm HIV-1 seropositivity or HIV-1 exposure Severe acute malnutrition Possible tuberculosis (coughing for more than 14 days) Severe anemia (hemoglobin <8.0 g/dL) Severe malaria Known allergy to penicillin or amoxicillin Receipt of an antibiotic treatment in the 48 hours prior to the study Hospitalized within 14 days prior to the study Living outside the study area Any medical or psychosocial condition or circumstance that, in the opinion of the investigators, would interfere with the conduct of the study or for which study participation might jeopardize the child’s health Any non-pneumonia acute medical illness which requires antibiotic treatment per local standard of care Participation in a clinical study of another investigational product within 12 weeks prior to randomization or planning to begin participation during this study Prior participation in the study during a previous pneumonia diagnosis
Treatment failure	
Anytime on or before Day 6	Severe respiratory distress Hypoxemia WHO IMCI danger signs Missing >3 study drug doses due to vomiting Change in antibiotics prescribed by a study clinician Prolonged hospitalization or re-admission due to pneumonia Death
At or after initial hospitalization discharge (between 42 and 60 hours post-enrollment)	Axillary temperature >38ºC with chest indrawing
On Day 6	Axillary temperature >38ºC Chest indrawing
Relapse	
After Day 6	Recurrence of signs of chest-indrawing pneumonia, severe respiratory distress (e.g., grunting, nasal flaring, head nodding, or severe chest indrawing) or severe disease

The study was conducted in accordance with International Conference on Harmonisation, Good Clinical Practice and the Declaration of Helsinki 2008, and was approved by Western Institutional Review Board, USA; College of Medicine Research and Ethics Committee, Blantyre, Malawi; and Malawi Pharmacy, Medicines and Poisons Board (Appendix 1: Protocol).

ASG and SM designed the study. TM, MP, CN, and AP gathered the data. RS, JH, and SM analyzed the data. All authors vouch for the data and analysis and decided to publish the paper. ASG, EN and SM wrote the first draft of the paper. There were no confidentiality agreements between funder, sponsor, or any involved institutions.

### Procedures

On Day 1, eligible children were randomized and enrolled, double-blinded, in a 1:1 ratio to receive either 3-day twice-daily amoxicillin dispersible tablets (DT) followed by 2-day twice-daily placebo DT (intervention) or 5-day twice-daily amoxicillin DT (control). High-dose oral amoxicillin was provided in 250mg DT in 2 divided doses based on age bands (500mg/day for children 2-11 months, 1000mg/day for 12-35 months, and 1,500mg/day for 36-59 months of age), current WHO-recommended therapy for HIV-uninfected children.^2^ Study drugs were identical in appearance, smell, taste, dispersion activity and packaging. Randomization was stratified by age groups (2-11, 12-35 and 36-59 months) using blocks of size 2, 4 and 6. Other than unblinded biostatisticians, pharmacists, monitor, and data and safety monitoring board (DSMB) members, everyone else on the study team was blinded to each child’s assigned treatment group.

Enrollment was conducted solely at KCH initially (phase 1), and then transitioned (September 20, 2016) to BDH (phase 2) after KCH introduced user fees which reduced patient volumes. BDH enrollees were transferred to KCH for additional evaluation and admission. To maximize safety, most enrollees were hospitalized for 2 days and discharged on Day 3 if no TF criteria (Table 1) were present.

Enrolled children were evaluated on Days 2 (while hospitalized), 4, 6, and 14 in clinic or home. During follow-up, all children were assessed for TF or relapse and study drug adherence at all scheduled and unscheduled visits. Most TF or relapse cases were hospitalized and treated with intravenous antibiotics. Once on intravenous or other second-line antibiotics, the child was considered non-adherent to randomized treatment.

### Outcomes

The primary endpoint was the proportion of children with Day 6 TF (Panel). Secondary endpoints included proportions of children with relapse (Days 7-14 among children without TF before or on Day 6), and with Day 6 TF or relapse by Day 14. Four of 6 prespecified subgroups are reported with respect to TF by age groups, malnutrition, malaria, and very fast breathing for age. Prespecified subgroups of low oxygen saturation (n=10) and wheeze (n=49) are not reported due to small numbers.

All adverse events were assessed and managed per KCH standard clinical practice, documented, and followed and treated until resolution or stabilization. All serious adverse events were reported to the study safety team for review within 24 hours.

### Statistical analysis

A relative non-inferiority margin of 1.5 times the TF rate in the 5-day amoxicillin group was chosen based on an anticipated TF rate in the 5-day group of 8%. This non-inferiority margin, 50% higher TF rate in the 3-day compared to the 5-day group, was chosen after extensive discussions among the investigators and with external experts regarding what TF rate might be acceptable to clinicians for the 3-day compared to the 5-day group, considering the anticipated potential TF rate in the 5-day group and potential for enrollment into the study. Initially adjusting for 2 formal interim analyses (with O’Brien-Fleming boundary for early noninferiority^13^ and Pocock boundary for early inferiority^14^), enrolling 2,000 children (1,000 per group) provided 88.1% power if the TF rate was equal in both groups at 8%, and 64.8% power if the TF rate was 4% in both groups. A potential increase in sample size was considered during planning of the study in case the overall TF rate was much lower than the anticipated 8%. After the second formal interim analysis, it was clear that the overall TF rate was less than 6%. To maintain a power (with equal TF rates) of 80% or higher, the maximum sample size was increased to 3,000 children (1,500 per group), and a third formal interim analysis was performed after a little more than 2,000 children were enrolled. The decision to increase the sample size was made by blinded study investigators after consultation with the funding agency. With increase in maximum sample size (and assuming equal TF rates in each group), the study had 84.8% and 89.8% power for 5% and 6% TF rates, respectively. Power calculations took into account a drop-out rate of 5% and assumed a 1-sided alpha of 0.025 for a test of a difference in proportions. Primary analyses were performed based on the intent-to-treat principle of complete cases using linear regression adjusted for age groups, study phase and sex, and using robust standard errors based on the Huber-White sandwich estimator.^15,16^ Justified because the sample size was sufficiently large, linear regression was used for this binary outcome to model differences in rates.^17^ Estimates for treatment differences for prespecified subgroups are reported with individual 95% confidence intervals (CIs) without adjustment for multiple comparisons. No post-hoc subgroup analyses were performed. The independent DSMB considered formal stopping boundaries during their interim reviews, but decided not to follow them, but rather, treat them as guiding only. Thus, the primary analysis was not adjusted for interim monitoring. Sensitivity analyses were performed using multiple imputations and tipping point analyses.^18^ For multiple imputations, a hot-deck approach (20 imputations) was used considering a match on at least 3 of the following 5 factors: age (2-11, 12-35, 36-50 months), sex, mother’s education (none, primary, secondary/tertiary), number of children in the home (1, 2, 3, 4+), and number of amoxicillin doses taken (≤4, 5-7, 8-9, 10). Analyses of secondary endpoints used robust standard errors unadjusted for interim analyses or other factors. Of 6 prespecified subgroup analyses, 4 are reported.

## RESULTS

**Figure 1:**
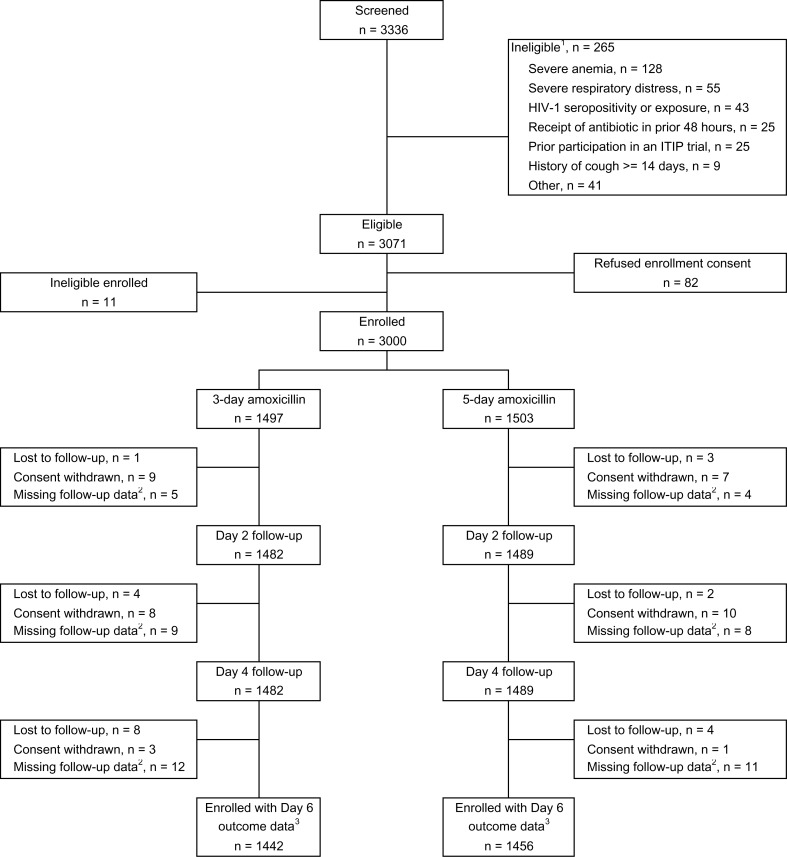
Consort diagram by treatment group ^1^Children may be ineligible for more than one reason. ^2^Missing follow-up data may be due to missed visits or visits occurring outside visit windows. ^3^Missing follow-up data n’s do not add up because some children had missing follow-up data for either Day 2 or Day 4 or both, but had outcome data available for Day 6.

Enrollment started March 29, 2016 with formal interim analyses after 1/3, 2/3 and slightly above the original maximum planned enrollment, and the last visit was completed April 14, 2019. In total, 3336 children were screened, of which 265 were ineligible (Figure 1). Of these 265, 11 were enrolled, and 82 were eligible but refused enrollment consent. A total of 3000 children were enrolled with 1497 receiving 3-day and 1503 receiving 5-day amoxicillin. Primary outcome was available for 1442 (96.3%) and 1456 (96.9%) children in the 3-and 5-day groups respectively. Baseline characteristics were similar between groups (Table 2).

**Table 2:** Child characteristics at enrollment by treatment group

	3-day amoxicillin (n=1497)	5-day amoxicillin (n=1503)	Overall (n=3000)
Age (months)	1497	1503	3000
2-11	867 (57.9%)	869 (57.8%)	1736 (57.9%)
12-35	509 (34.0%)	514 (34.2%)	1023 (34.1%)
36-59	121 (8.1%)	120 (8.0%)	241 (8.0%)
Sex	1497	1503	3000
Male	833 (55.6%)	820 (54.6%)	1653 (55.1%)
Female	664 (44.4%)	683 (45.4%)	1347 (44.9%)
Height/weight Z-score^1^	1497	1503	3000
<-3	0 (0%)	0 (0%)	0 (0%)
-2 to -3 >-2	10 (0.7%) 1487 (99.3%)	14 (0.9%) 1489 (99.1%)	24 (0.8%) 2976 (99.2%)
Mid-upper arm circumference (cm)^1^	1497	1503	3000
<11.5	0 (0%)	0 (0%)	0 (0%)
11.5-13.5	321 (21.4%)	304 (20.2%)	625 (20.8%)
>13.5	1176 (78.6%)	1199 (79.8%)	2375 (79.2%)
Respiratory rate (breaths/min)^2^	1497	1503	3000
Age 2-11 months	867	869	1736
<50	300 (34.6%)	303 (34.9%)	603 (34.7%)
50-59	361 (41.6%)	371 (45.2%)	754 (43.4%)
≥60	206 (23.8%)	173 (19.9%)	379 (21.8%)
Age 12-59 months	630	634	1264
<40	160 (25.4%)	164 (25.9%)	324 (25.6%)
40-49	251 (39.8%)	262 (41.3%)	513 (40.6%)
≥50	219 (34.8%)	208 (32.8%)	427 (33.8%)
Oxygen saturation (%)^3^	1497	1503	3000
<90	0 (0%)	0 (0%)	0 (0%)
90-92	5 (0.3%)	7 (0.5%)	12 (0.4%)
≥93	1492 (99.7%)	1496 (99.5%)	2988 (99.6%)
Axillary temperature (°C)^2^	1497	1503	3000
<38	1020 (68.1%)	1054 (70.1%)	2074 (69.1%)
≥38	477 (31.9%)	449 (29.9%)	926 (30.9%)
Mean heart rate beats/min^1^ (SD)	146.9 (17.4)	146.1 (17.1)	146.5 (17.3)
Pneumococcal conjugate vaccine (PCV13)	1497	1503	3000
Received age-appropriate number of doses^4^	942 (62.9%)	952 (63.3%)	1894 (63.1%)
Received <age-appropriate number of doses or unknown	555 (37.1%)	551 (36.7%)	1106 (36.9%)
Pentavalent vaccine	1497	1503	3000
Received age-appropriate number of doses^4^	949 (63.4%)	952 (63.3%)	1901 (63.4%)
Received <age-appropriate number of doses or unknown	548 (36.6%)	551 (36.7%)	1099 (36.6%)
**Caregiver assessment at enrollment**
Fever^1^	1497	1503	3000
Yes	1172 (78.3%)	1148 (76.4%)	2320 (77.3%)
Mean number of days (SD)	2.4 (1.1)	2.5 (1.2)	2.4 (1.1)
Cough^1^	1497	1503	3000
Yes	1479 (98.8%)	1496 (99.5%)	2975 (99.2%)
Mean number of days (SD)	2.6 (1.3)	2.6 (1.3)	2.6 (1.3)
Difficult breathing^1^	1491	1498	2989
Yes	585 (39.2%)	552 (36.8%)	1137 (38.0%)
Mean number of days (SD)	2.4 (1.1)	2.4 (1.1)	2.4 (1.1)

Data are n (%) or mean (standard deviation).

1 Data not available for all randomized children.

2 Larger value between screening and enrollment visits.

3 Smaller value between screening and enrollment visits.

4 Three doses for children aged 14 weeks and older; 2 doses for children aged 10 weeks up to 14 weeks; and 1 dose for children aged 6 weeks up to 10 weeks.

By Day 6, 3-day recipients had a TF rate of 5.9% (85/1442 with Day 6 outcome) and 5-day recipients 5.2% (75/1456), resulting in an adjusted absolute TF rate difference (intent-to-treat complete cases primary analysis) of 0.75% (95%CI -0.92%,2.41%, compared to a non-inferiority upper limit of 2.58%) (Table 3). Among children without TFby Day6,91/1326(6.9%)had relapse byDay14 in the3-day group,comparedwith 79/1354(5.8%)in the5-daygroup,representing an absolute differencein the relapse rate of 1.0 (95%CI -0.8%,2.9%).

**Table 3:** Outcomes by treatment group

	3-day amoxicillin (n=1497)	5-day amoxicillin (n=1503)	Difference (95% CI)
Primary			
Treatment failure on or prior to Day 6^1^	85 / 1442 (5.9%)	75 / 1456 (5.2%)	0.75% (-0.92%, 2.41%)
Secondary - *a priori*			
Relapse on or prior to Day 14 if cured by Day 6^2^	91 / 1326 (6.9%)	79 / 1354 (5.8%)	1.0% (-0.8% to 2.9%)
Treatment failure or relapse on or prior to Day 14	176 / 1411 (12.5%)	154 / 1429 (10.8%)	1.7% (-0.7% to 4.1%)
Multiple imputation for any missing primary outcome data due to withdrawal or loss to follow-up^3^	Imputed for n=55	Imputed for n=47	0.8% (-0.9% to 2.4%)
Treatment failure subgroups - *a priori*			
Age (months) groups	1442	1456	
2-11	57 / 832 (6.9%)	46 / 842 (5.5%)	1.4% (-0.9% to 3.7%)
12-35	23 / 490 (4.7%)	24 / 498 (4.8%)	-0.1% (-2.8% to 2.5%)
36-59	5 / 120 (4.2%)	5 / 116 (4.3%)	-0.1% (-5.3% to 5.0%)
Mid-upper arm circumference (cm)^4^	1442	1456	
<11.5	0 / 0	0 / 0	
11.5-13.5	25 / 309 (8.1%)	17 / 297 (5.7%)	2.4% (-1.7% to 6.4%)
>13.5	60 / 1133 (5.3%)	58 / 1159 (5.0%)	0.3% (-1.5% to 2.1%)
Malaria	1442	1456	
Positive	4 / 127 (3.1%)	5 / 136 (3.7%)	-0.5% (-4.9% to 3.9%)
Negative	81 / 1315 (6.2%)	70 / 1320 (5.3%)	0.9% (-0.9% to 2.6%)
Very fast breathing for age	1442	1456	
Positive	5 / 68 (7.4%)	5 / 59 (8.5%)	-1.1 (-10.6 to 8.3)
Negative	80 / 1374 (5.8%)	70 / 1397 (5%)	0.8 (-0.9 to 2.5)

Data are n (%). 95% CI=95% confidence interval. Some results may appear inconsistent due to rounding.

1 Difference and 95% CI adjusted for age, sex and phase.

2 Of those without treatment failure on or prior to Day 6.

3 Covariates used in imputation: treatment group, age group, sex, mother's education level, number of children in the home and number of doses taken.

4 Data not available for all randomized children.

Prior to Day 4, both the 3-and 5-day groups were receiving amoxicillin, and as such, we would expect the TF rate prior to Day 4 to be the same. The TF rates prior to Day 4 in the 3-and 5-day groups were 2.3% (33/1442) and 2.3% (33/1456), respectively (post-hoc descriptive unadjusted). During Days 4 and 5, the 3-day group was receiving placebo whereas the 5-day group continued to receive amoxicillin. The TF rates for Days 4 through 6 in the 3-and 5-day groups were 3.6% (52/1442) and 2.9% (42/1456), respectively.

When considering both TF before or by Day 6 and relapse by Day 14, 176/1411 (12.5%) in the 3-day groupand 154/1429(10.8%) in the5-daygroupmet criteria(absolutedifferenceof1.7%, 95%CI 0.7%,4.1%). Additional secondary outcomes results are detailed in Table 3. The TF rate was generally consistent across prespecified subgroupsdefined byagegroups, malnutrition,malaria, and very fast breathing for age. Most 95% CIs for the subgroups did not exclude a 1.5 non-inferiority margin and any adjustment for multiple comparisons would have resulted in all 95% CIs including the non-inferiority margin. The amount of missing primary outcome data was low (overall n=102, 3.4%; n=55 and n=47 in the3-and 5-daygroupsrespectively).Estimates derived from multiple imputationsfor missing outcome data were similar to the complete case analysis. When considering a tipping point analysis, we failed to conclude non-inferiority only if there were at least 3 additional children with TF among children in the 3day group compared to children in the 5-day group among those who have missing data. If the same TF rates observed for the completedata appliedto the missing data, theexpected averagedifferenceis 1.2 individuals (55*5.9%-47*5.2% = 1.2). Assuch, we would have needed to observe a largerdifference amongthe missing data (e.g.,3out of 55,and0out of 47) in order tofail to conclude non-inferiority.If primary resultswould have been adjusted for sequential monitoring, the conclusion of non-inferiority remains the same.

Thepercentof children with at least1 seriousadverse eventbetween enrollment andDay14 was9.8% in the 3-day group compared to 8.8% in the 5-day group (Table 4). There was 1 (0.1%) death due to pneumoniain the3-daygroup,and2 (0.1%) deaths, 1 due to pneumonia and 1dueto acute gastroenteritis,in the5-daygroup.

**Table 4:** Serious and common non-serious adverse events by treatment group

	3-day amoxicillin (n=1497) n (%)	5-day amoxicillin (n=1503) n (%)	Overall (n=3000) n (%)
Children with at least 1 serious adverse event1,2	147 (9.8%)	132 (8.8%)	279 (9.3%)
Children with at least 1 non-serious adverse event1,2	395 (26.3%)	455 (30.3%)	849 (28.3%)
Serious adverse events (can be multiple events of the same or different type per child)			
Pneumonia	135 (9%)	118 (7.9%)	253 (8.4%)
Chest-indrawing pneumonia	61 (4.1%)	49 (3.3%)	110 (3.7%)
Danger sign pneumonia	49 (3.3%)	51 (3.4%)	100 (3.3%)
Fast-breathing pneumonia3	17 (1.1%)	14 (0.9%)	31 (1.0%)
Chest radiograph-confirmed pneumonia4	7 (0.5%)	3 (0.2%)	10 (0.3%)
Pneumonia not otherwise specified	1 (0.1%)	1 (0.1%)	2 (0.1%)
Non-pneumonia	20 (1.3%)	15 (1%)	35 (1.2%)
Gastroenteritis	8 (0.5%)	6 (0.4%)	14 (0.5%)
Fever	3 (0.2%)	5 (0.3%)	8 (0.3%)
Malaria	1 (0.1%)	2 (0.1%)	3 (0.1%)
Meningitis	3 (0.2%)	0 (0%)	3 (0.1%)
Otitis media	2 (0.1%)	0 (0%)	2 (0.1%)
Conjunctivitis	1 (0.1%)	0 (0%)	1 (0%)
Edema	0 (0%)	1 (0.1%)	1 (0%)
Febrile seizure	1 (0.1%)	0 (0%)	1 (0%)
Rectal prolapse	1 (0.1%)	0 (0%)	1 (0%)
Vomiting	0 (0%)	1 (0.1%)	1 (0%)
Common non-serious adverse events (can be multiple events of the same or different type per child)			
Gastroenteritis	176 (11.7%)	223 (14.9%)	399 (13.3%)
Upper respiratory infection	113 (7.5%)	114 (7.6%)	227 (7.6%)
Rash	32 (2.1%)	50 (3.3%)	79 (2.6%)
Conjunctivitis	21 (1.4%)	20 (1.3%)	41 (1.4%)
Rhinitis	22 (1.5%)	15 (1%)	37 (1.2%)
Otitis media	13 (0.9%)	21 (1.4%)	34 (1.1%)
Eczema	15 (1.0%)	17 (1.1%)	32 (1.1%)
Oral candidiasis	13 (0.9%)	15 (1.0%)	28 (0.9%)

1 Occurring any time after study drug is administered to child up to 14 days after enrollment.

2 Children may have more than 1 serious and/or non-serious adverse event. 337 occurred on or prior to Day 6 and were treatment failures while the remaining occurred after Day 6 and thus were considered relapses. 4The chest radiograph-confirmed pneumonia serious adverse events did not demonstrate fast breathing, chest indrawing, or any danger signs; however, pneumonia was diagnosed through positive chest radiographs.

Caregiver-reported adherencewas high with91.6% reporting adherencewith all doses in the3-day groupand 91.8% reportingadherencewith all dosesin the5-daygroup.

## DISCUSSION

We evaluated 3-versus 5-day oral amoxicillin treatment among 3000 HIV-uninfected children aged 2-59 months presenting with WHO–defined chest-indrawing pneumonia in a malaria-endemic region of Malawi. Our results demonstrated that those children who received 3-day amoxicillin had a non-inferior TF rate on or before Day 6 compared to those who received 5-day amoxicillin. By Day 14, non-inferiority appeared to continue.

This study suggests that 3-day amoxicillin is not substantively worse than 5-day amoxicillin for treatment of chest-indrawing pneumonia among HIV-uninfected children. Keeping in mind both individual and health system benefits of a shorter course of antibiotic therapy, and that WHO already recommends 3-day amoxicillin for treatment of fast-breathing pneumonia^2,5-7^ it appears that 3-day amoxicillin for children with chest-indrawing pneumonia might be sufficient. Currently, WHO recommends a 5-day course of twice-daily high-dose oral amoxicillin to treat chest-indrawing in a child with cough or difficulty breathing.^2,19^ However, the findings of this study may allow for harmonization and simplification of treatment courses for both fast-breathing and chest-indrawing pneumonia to be 3 days among HIV-uninfected children. A study from Pakistan found that in cases of chest-indrawing pneumonia without underlying complications, home treatment with a short-course of high-dose oral amoxicillin was preferable to parenteral treatment because of the associated reduction in referral, admission, and treatment costs.^19^ Home treatment of chest-indrawing pneumonia with oral amoxicillin is effective across communities and geographic regions.^20-22^ In contrast to low-resource settings, in high-resource settings, criteria for diagnosing pneumonia often require chest radiographic confirmation, especially in hospitalized children.^23^ Yet, little evidence exists to dictate treatment duration.^11^ Of note, in a very small study from Israel, a 3-day course of oral high-dose amoxicillin was associated with a high TF rate of 40% (4/10) among children with radiograph-confirmed pneumonia.^24^

Poor adherence to antibiotics has been associated with TF in WHO-defined clinical pneumonia.^25,26^ Improving adherence with shorter course treatment could improve outcomes in children with chest-indrawing pneumonia while also minimizing adverse drug effects, costs, and the emergence of antimicrobial resistance.^7,25,26^

### Limitations

Limitations in our study included strict inclusion and exclusion criteria, absence of laboratory or radiology testing, and close monitoring and follow-up, which limits the generalizability of our results to routine programmatic care settings. Notably, severe disease was excluded which limits applicability. Pneumonia is frequently considered a single entity, rather than a clinical syndrome encompassing several underlying factors. This makes interpretation of results challenging. Without etiological information, we could only note the effect of the intervention on the clinical syndrome of pneumonia, which is an approach consistent with non-trial conditions relevant to pediatric care in low-resource settings.

Follow-up care and monitoring of enrolled children generally exceeded local standards of care and thus, the TF rate may have been influenced by both the high quality of care provided and by the high awareness and vigilance for identifying TF. It may be that those identified as failing treatment would have recovered without a longer course of antibiotics had we taken a watchful waiting approach and not intervened with antibiotic treatment. However, opportunities for follow-up and access to care are often issues in low-resource settings. In addition, treatment approaches vary widely between countries and regions. Routine pediatric HIV testing included in this study protocol, while recommended, is not rigorously implemented during routine care in low-resource HIV-endemic settings.^27^ In areas where pneumococcal immunization coverage is lower, HIV endemicity is high, or where severe acute malnutrition or other predisposing conditions for bacterial disease is common, it may be reasonable to expect a higher TF rate among those not treated with a longer course of antibiotics. As such, our results might not be generalizable across different regions, settings, or non-trial conditions. Specifically, the percentage of TF or relapse observed in this study might be underestimating true TF and relapse rates experienced during non-trial conditions.

## CONCLUSIONS

Despite pneumonia being a common and deadly illness, optimal duration of antibiotic treatment for community-acquired pediatric pneumonia has not yet been established. In this population in Malawi, 3-day was non-inferior to 5-day amoxicillin treatment among HIVuninfected children with chest-indrawing pneumonia. In considering policy changes regarding duration of amoxicillin treatment for chest-indrawing pneumonia, further research may be needed to see if these results can be replicated in other low-resource regions and pediatric populations.

## Funding

This work was supported by a grant from the Bill and Melinda Gates Foundation [OPP1105080].

## Declaration of interests

ASG and EN report grants from the Bill & Melinda Gates Foundation. TM, MP, RS, JH, CN, AP and SM report grants from Save the Children Federation, Inc. EDM reports grants from the Bill & Melinda Gates Foundation, GlaxoSmithKline, and the National Institute of Environmental Health Sciences. RI is employed by the Bill & Melinda Gates Foundation. SM reports grants from the National Heart, Lung, and Blood Institute, the Department of Defense, the National Institute of Allergy and Infectious Diseases, and personal fees from various academic and for-profit entities, Novo Nordisk, and the National Institute of Neurological Disorders and Stroke.

## References

[1] Liu L, Oza S, Hogan D, et al. Global, regional, and national causes of child mortality in 2000-13, with projections to inform post-2015 priorities: an updated systematic analysis. Lancet 2015;385:430-40. 25280870 10.1016/S0140-6736(14)61698-6

[2] WHO Revised WHO classification and treatment of pneumonia in children at health facilities: evidence summaries. Geneva: World Health Organization; 2014. 25535631

[3] WHO Integrated management of childhood illness: chart booklet. Geneva: World Health Organization; 2014.

[4] Lassi ZS, Das JK, Haider SW, Salam RA, Qazi SA, Bhutta ZA Systematic review on antibiotic therapy for pneumonia in children between 2 and 59 months of age. Arch Dis Child 2014;99:687-93. 24431417 10.1136/archdischild-2013-304023

[5] Haider BA, Saeed MA, Bhutta ZA Short-course versus long-course antibiotic therapy for non-severe community-acquired pneumonia in children aged 2 months to 59 months. Cochrane Database Syst Rev 2008:CD005976. 18425930 10.1002/14651858.CD005976.pub2

[6] Pakistan Multicentre Amoxycillin Short Course Therapy pneumonia study g. Clinical efficacy of 3 days versus 5 days of oral amoxicillin for treatment of childhood pneumonia: a multicentre double-blind trial. Lancet 2002;360:835-41. 12243918 10.1016/S0140-6736(02)09994-4

[7] Agarwal G, Awasthi S, Kabra SK, et al. Three day versus five day treatment with amoxicillin for non-severe pneumonia in young children: a multicentre randomised controlled trial. BMJ 2004;328:791. 15070633 10.1136/bmj.38049.490255.DE PMC383371

[8] Lassi ZS, Imdad A, Bhutta ZA Short-course versus long-course intravenous therapy with the same antibiotic for severe community-acquired pneumonia in children aged two months to 59 months. Cochrane Database Syst Rev 2017;10:CD008032. 29020436 10.1002/14651858.CD008032.pub3 PMC6485461

[9] McMullan BJ, Andresen D, Blyth CC, et al. Antibiotic duration and timing of the switch from intravenous to oral route for bacterial infections in children: systematic review and guidelines. Lancet Infect Dis 2016;16:e139-52. 27321363 10.1016/S1473-3099(16)30024-X

[10] Das RR, Singh M Treatment of severe community-acquired pneumonia with oral amoxicillin in under-five children in developing country: a systematic review. PLoS One 2013;8:e66232. 23825532 10.1371/journal.pone.0066232 PMC3692509

[11] Grimwood K, Fong SM, Ooi MH, Nathan AM, Chang AB Antibiotics in childhood pneumonia: how long is long enough? Pneumonia (Nathan) 2016;8:6. 28702286 10.1186/s41479-016-0006-x PMC5469190

[12] Ginsburg AS, May SJ, Nkwopara E, et al. Methods for conducting a double-blind randomized controlled clinical trial of three days versus five days of amoxicillin dispersible tablets for chest indrawing childhood pneumonia among children two to 59 months of age in Lilongwe, Malawi: a study protocol. BMC Infect Dis 2018;18:476. 30241517 10.1186/s12879-018-3379-z PMC6151015

[13] O'Brien PC, Fleming TR A multiple testing procedure for clinical trials. Biometrics 1979;35:549-56. 497341

[14] Pocock SJ Group sequential methods in the design and analysis of clinical trials. Biometrika 1977;64:191-9.

[15] Huber PJ The behavior of maximum likelihood estimates under nonstandard conditions. Proceedings of the Fifth Berkeley Symposium on Mathematical Statistics and Probability. Berkeley: University of California Press; 1967:221-33.

[16] White HL Jr . Maximum likelihood estimation of misspecified models. Econometrica 1982;50:1-25.

[17] Lumley T, Diehr P, Emerson S, Chen L The importance of the normality assumption in large public health data sets. Annu Rev Public Health 2002;23:151-69. 11910059 10.1146/annurev.publhealth.23.100901.140546

[18] Andridge RR, Little RJ A Review of Hot Deck Imputation for Survey Non-response. Int Stat Rev 2010;78:40-64. 21743766 10.1111/j.1751-5823.2010.00103.x PMC3130338

[19] Hazir T, Fox LM, Nisar YB, et al. Ambulatory short-course high-dose oral amoxicillin for treatment of severe pneumonia in children: a randomised equivalency trial. Lancet 2008;371:49-56. 18177775 10.1016/S0140-6736(08)60071-9

[20] Addo-Yobo E, Anh DD, El-Sayed HF, et al. Outpatient treatment of children with severe pneumonia with oral amoxicillin in four countries: the MASS study. Trop Med Int Health 2011;16:9951006. 10.1111/j.1365-3156.2011.02787.x PMC3154370 21545381

[21] Bari A, Sadruddin S, Khan A, et al. Community case management of severe pneumonia with oral amoxicillin in children aged 2-59 months in Haripur district, Pakistan: a cluster randomised trial. Lancet 2011;378:1796-803. 22078721 10.1016/S0140-6736(11)61140-9 PMC3685294

[22] Soofi S, Ahmed S, Fox MP, et al. Effectiveness of community case management of severe pneumonia with oral amoxicillin in children aged 2-59 months in Matiari district, rural Pakistan: a cluster-randomised controlled trial. Lancet 2012;379:729-37. 22285055 10.1016/S0140-6736(11)61714-5

[23] Nascimento-Carvalho CM, Madhi SA, O'Brien KL Review of guidelines for evidence-based management for childhood community-acquired pneumonia in under-5 years from developed and developing countries. Pediatr Infect Dis J 2013;32:1281-2. 24141800 10.1097/INF.0b013e3182a4dcfa

[24] Greenberg D, Givon-Lavi N, Sadaka Y, Ben-Shimol S, Bar-Ziv J, Dagan R Short-course antibiotic treatment for community-acquired alveolar pneumonia in ambulatory children: a double-blind, randomized, placebo-controlled trial. Pediatr Infect Dis J 2014;33:136-42. 23989106 10.1097/INF.0000000000000023

[25] Nightingale R, Colbourn T, Mukanga D, et al. Non-adherence to community oral-antibiotic treatment in children with fast-breathing pneumonia in Malawi-secondary analysis of a prospective cohort study. Pneumonia (Nathan) 2016;8:21. 28702300 10.1186/s41479-016-0024-8 PMC5471995

[26] King C, Nightingale R, Phiri T, et al. Non-adherence to oral antibiotics for community paediatric pneumonia treatment in Malawi - A qualitative investigation. PLoS One 2018;13:e0206404. 30379968 10.1371/journal.pone.0206404 PMC6209296

[27] Theodoratou E, McAllister DA, Reed C, et al. Global, regional, and national estimates of pneumonia burden in HIV-infected children in 2010: a meta-analysis and modelling study. Lancet Infect Dis 2014;14:1250-8. 25455992 10.1016/S1473-3099(14)70990-9 PMC4242006

